# Supplementary material for: 24-Epibrassinolide-Succinic Acid Conjugate Is Involved in the Acclimation of Rape Plants to Salt Stress
Source: Plants (Basel). 2025 Nov 6;14(21):3404. doi: 10.3390/plants14213404 (PMC12610500; doi:10.3390/plants14213404)
Supplement: Supplementary file 1 [file plants-14-03404-s001.zip › plants-3921484-supplementary.pdf]

Supplementary Materials

Methods S1: 2,3,22,23-Tetrahemisuccinate of 24-epibrassinolide is a stable compound. Storage at 0-5°C for one year does not alter its spectral characteristics. UV spectrum (λmax, nm (ε), MeOH): 2960, 2875, 1740, 1710, 1410, 1370, 1170. 1H NMR spectrum (500 MHz, C5D5N, δ, ppm, J, Hz): 0.76 (s, 3H, H18), 0.88 (d, J 7.0, 3H, H28), 0.93 (d, J 7.0, 3H, H27), 0.99 (d, J 7.0, 3H, H26), 1.02 (s, 3H, H19), 1.05 (d, J 6.7, 3H, H21), 2.64–2.71 (m, 16H, HOOC–CH2CH2–), 4.93 (d, J 11.2, 1H, H2), 5.20 (dd, J 8.7, 3.3, 1H, H23), 5.35 (d, J 8.6, 1H, H22), 5.44 (s, 1H, H3). 13C NMR spectrum (125 MHz, C 5D5N, δ, ppm): 11.3 (CH3, C28), 11.8 (CH3, C18), 13.6 (C, C21), 15.6 (CH3, C19), 17.8 (q), 22.9 (q), 23.3 (CH2, C11), 25.6 (CH2, C15), 28.2 (d), 28.7 (CH2, C16), 28.9 (CH2, C4), 29.5, 29.7, 29.9, 30.0, 30.1, 30.2, 30.3, 30.5 (8CH2, Csuc.acid.), 38.9 (CH, C8), 39.3 (C, C10), 39.9 (CH2, C12), 40.1 (CH, C20), 40.5 (CH, C24), 40.6 (CH2, C1), 43.0 (CH, C5), 43.6 (C, C13), 52.1 (CH, C14), 53.7 (CH, C17), 59.6 (CH, C9), 69.9 (CH, C3), 70.7 (CH, C2), 71.7 (CH2, C7), 76.1 (CH, C22), 78.9 (CH, C23), 173.3, 173.4, 173.7, 174.0, 175.7, 175.9, 176.0, 176.1 (8C, C=O, Csuc.acid.), 178.3 (C, C6). Mass spectrum (APCI), m/z (Irel,%): 881 [M + H]+ (60), 903 [M + Na]+ (100). HRMS (ESI) m/z: found: 903.3972 [M + Na]+; calculated for C44H64O18Na: 903.3990.

Table S1. Effect of plants pretreatment (4 hours) with succinic acid on rapeseed growth under optimal conditions

Treatment	Hypocotyl length (cm)	Stem length (cm)	Root length (cm)	Total leaf area (cm^2)	Total wet weight of the plant (g)
Control	4.10 ± 0.15^a	3.53 ± 0.31^a	17.93 ± 1.02^a	118.01 ± 11.84^a	4.43 ± 0.43^a
10 nM	3.95 ± 0.10^a	3.42 ± 0.21^a	16.92 ± 0.69^a	117.69 ± 6.01^a	4.39 ± 0.40^a
40 nM	3.83 ± 0.15^a	3.33 ± 0.21^a	16.89 ± 0.76^a	116.69 ± 4.68^a	4.10 ± 0.25^a
1 μM	3.73 ± 0.15^b	3.23 ± 0.21^a	14.10 ± 0.68^b	102.13 ± 8.15^a	4.14 ± 0.30^a
0.01 mM	3.80 ± 0.20^a	3.03 ± 0.29^a	15.23 ± 0.87^b	82.99 ± 5.98^b	3.90 ± 0.32^a
0.1 mM	3.97 ± 0.13^a	4.43 ± 0.19^b	16.87 ± 0.77^a	127.28 ± 5.83^a	5.85 ± 0.43^b
1 mM	3.87 ± 0.12^a	3.37 ± 0.24^a	17.63 ± 0.88^a	109.46 ± 12.27^a	4.49 ± 0.45a
10 mM	3.90 ± 0.13^a	3.73 ± 0.26^a	18.07 ± 0.75^a	118.69 ± 6.61^a	5.66 ± 0.31^b

Values are given as the mean ±SD for each treatment. Values not sharing a common or same alphabet letter (a-b), and they differ significantly at $p < 0.05$ (Duncan's multiple range test).

Under optimal growing conditions, pretreatment (4 hours) of plants with succinic acid at low concentrations (10 and 40 nM) did not affect growth indices at the end of the experiment, whereas higher concentrations of succinic acid (0.1 mM, 10 mM) stimulated the accumulation of crude biomass of rapeseed plants by 28-32% relative to the control.

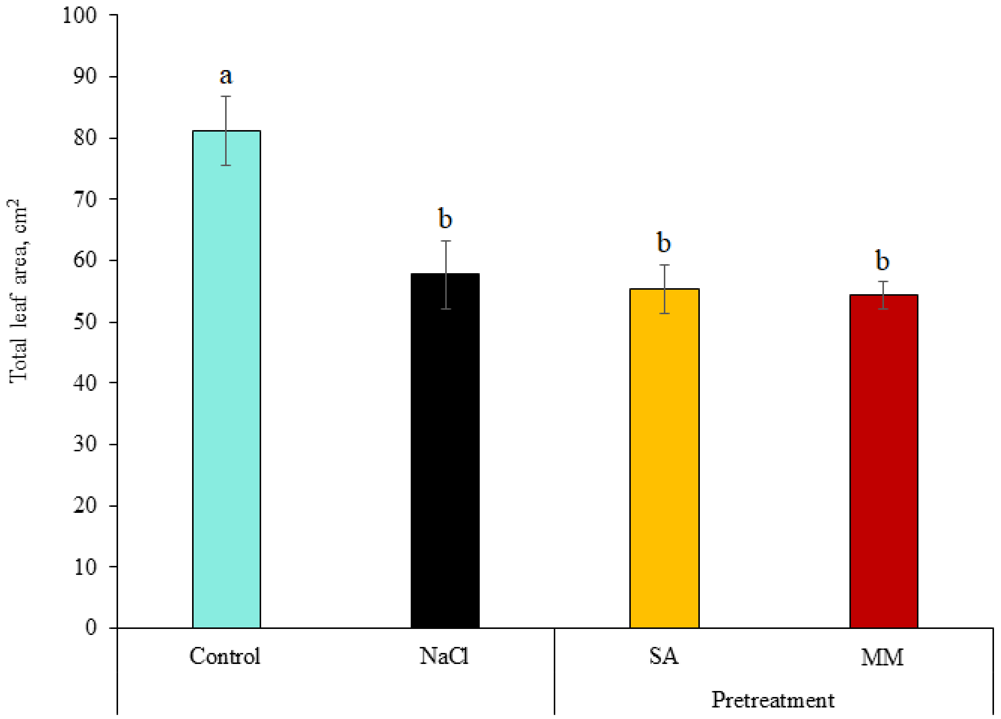

Figure S1. Effect of plants pretreatment (4 hours) with succinic acid (SA) and mechanical mixture (MM; SA + EBL) at a concentration of 10 nM against the background of subsequent chloride salinization (NaCl, 150 mM, 7 days) on the leaf surface area of rapeseed. Values are given as the mean ± SD for each treatment. Values are given as the mean ± SD for each treatment. Values not sharing a common or same alphabet letter (a-b), and they differ significantly at $p < 0.05$ (Duncan's multiple range test).

Pretreatment of rapeseed plants with exogenous succinic acid (SA) and mechanical mixture (MM; SA + EBL) at low concentrations (10 nM) did not significantly affect the growth of leaf area under salt stress (150 mM, 7 days).

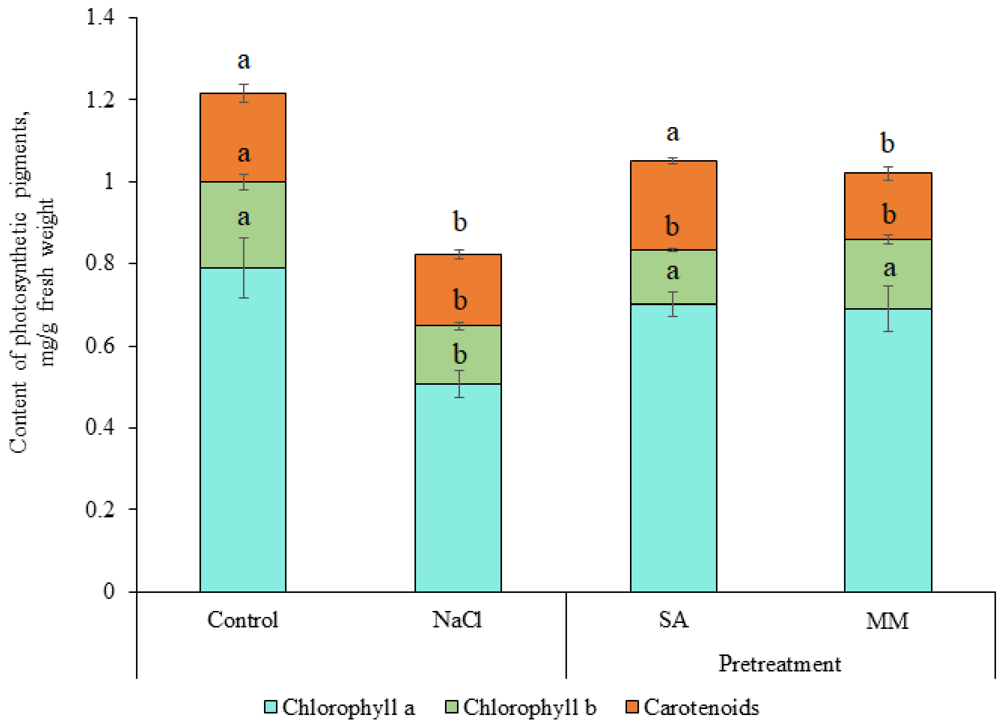

Figure S2. Effect of pretreatment of plants (4 hours) with succinic acid (SA) and mechanical mixture (MM; SA + EBL) at a concentration of 10 nM against the background of chloride salinity (NaCl, 150 mM, 7 days) on the content of the main photosynthetic pigments. Values are given as the mean ± SD for each treatment. Values not sharing a common or same alphabet letter (a-b), and they differ significantly at $p < 0.05$ (Duncan's multiple range test).

Treatment of plants with SA or a mechanical mixture (MM; SA + EBL) in low concentrations led to a decrease in the inhibitory effect of salt stress on the content of photosynthetic pigments by 15-25%, relative to the salt variant.
